# Prevalence of Aggressive Behavior in Greek Elementary School Settings from Teachers’ Perspectives

**DOI:** 10.3390/bs13050390

**Published:** 2023-05-08

**Authors:** Argyro Bourou, Effie Papageorgiou

**Affiliations:** Department of Biomedical Sciences, University of West Attica, 12243 Athens, Greece

**Keywords:** aggression, bullying, reactive, proactive, elementary schools, anger expression, school violence

## Abstract

The present study aims to estimate the prevalence of bullying in Greek elementary schools and to study the risk factors that lead to bullying episodes. A structured questionnaire was given to 221 teachers of elementary schools and 71 kindergarten teachers from urban and rural Greek schools. They were asked to note the forms and the frequency of aggressive behaviors that they had witnessed during the school years 2020–2021 and 2021–2022, as well as the sociodemographic characteristics of the aggressive children involved. Statistical analyses of the data were conducted, and the obtained results show that specific forms of aggression are significantly correlated with gender and low academic performance. In addition, there is no form of aggressive behavior that is associated with the perpetrator’s age, nationality or family status. Further, the results of the factor analysis revealed four dominant factors in the aggressive behavior observed by teachers. The forms of bullying and the prevailing factors of aggressive behavior that dominate in Greek school settings are reported in the present study. Furthermore, a novel evaluation tool for teachers could potentially be developed based on the results of the present study.

## 1. Introduction

Aggressive behavior in schools (both primary and secondary) is an essential and serious issue that has raised great concern among the literature. School bullying represents a form of aggression that takes place in the school environment. Specifically, bullying is defined as repeated aggressive behavior on the part of a perpetrator towards one or more of their classmates [1]. Another characteristic of school bullying is an imbalance of power between the perpetrator and the victim [2,3]. Cyberbullying signifies bulling expressed by electronic means and includes two additional characteristics: anonymity and publicity [4].

Aggression in a child can be distinguished into two forms, proactive or reactive, depending on whether there is an act that provokes the aggressive behavior [5,6]. According to Amad [7], reactive aggression occurs when a child reacts to a specific event (whether it actually happened or the child perceived something as a threat). It is characterized by outbursts of anger and high emotional charge. On the contrary, a child who shows proactive aggression uses violent behavior as a means to achieve their goals, whether they are aimed at dominating or forcing another child or acquiring an object.

School bullying also appears in various degrees and forms [8] and is categorized as physical or verbal bullying, social exclusion or cyberbullying [8,9,10].

### 1.1. Aggressor’s Characteristics

Bullies tend to have specific characteristics, according to Olweus [11], such as externalizing problems such as hostility, impulsivity and aggression not only against their peers, but also against their teachers or other adults. They also tend to dominate their peers, gain satisfaction from the show of strength and show less empathy for their victims. They also exhibit a lack of concern for safety, retaliatory attitudes and social–emotional problems [12]. Despite their overall aggressive conduct, they do not have low levels of popularity among their peers [2]. They are also negatively influenced by their peers or other social factors [13]. Aggressors frequently exhibit conduct disorder problems, as well as low self-esteem [14,15].

Many studies have examined gender and age differences among bullies. In particular, Craig [16] noted that male children were more physically aggressive than the comparison group, and at older ages, they were also more verbally aggressive. On the other hand, only at older ages did female bullies exhibit more physical and verbal aggression. It is also noted that girls are usually prone to relational/indirect aggression [2,17]. In many studies, it is stated that boys are more prone than girls to being bullies or victims of bullies [13,18]. Furthermore, Ostrov and Keating [19] observed preschoolers’ aggressive behavior and found that boys displayed more physical and verbal aggression during free play than girls. Similar findings are also reported in other studies concerning preschoolers [20] and Greek adolescents [21]. As far as cyberbullying is concerned, both girls and boys are involved to the same extent according to a study that was conducted in primary schools [22].

Card et al. [23] in their meta-analytic review of 148 studies, found that neither direct nor indirect forms of aggression are linked to the perpetrator’s age.

As far as the bully’s nationality is concerned, findings in the literature are contradictory. Specifically, Almeida et al. [24] indicated that immigrants are not involved in peer violence more often than their U.S. born peers. On the other hand, Smokowski et al. [25] studied Latino adolescents’ behavior and suggested that certain culture risk factors, such as perceived discrimination and acculturation conflicts, are associated with adolescent aggression.

According to Dodge [26], peer social rejection contributes significantly to a child’s aggressive behavior, especially the development of reactive aggression.

Children’s psychological or learning disorders also seem to influence their aggressive behavior [27,28]. According to Unnever & Cornell [29], middle-school students who are diagnosed with ADHD are more likely to act aggressively two or three times a month (13%) in comparison with other students (8%). In the same study, it is reported that students with ADHD bully their peers more often due to self-control difficulties. Moreover, children with ADHD are at risk for having aggressive reactions [30,31].

A bully’s family context is likely to play an important role in the appearance of aggressive behavior. Children that have been maltreated by their families (physical or sexual abuse) are more likely to behave aggressively toward other children [32,33,34]. On the other hand, mothers that adopt a positive parental style and are highly responsive to their children seem to rear less aggressive children [35]. Additionally, the development of a healthy parental environment counteracts the risk of aggression and violence in children [36]. In addition to this finding, Demaray and Malecki [37] noted that bullies and bullies/victims received less social support from their parents and teachers in comparison with the control group.

### 1.2. Prevalence of Bullying in Greek School Settings

Over the last two decades a significant number of studies have been conducted in Greek schools regarding bullying. Characteristically, Kalliotis [38] noted that the 30 per cent of the pupils of five Greek elementary schools have been bullied at least once in a school year. Pateraki & Houndoumandi [39] also found that 14.7% of the sample investigated (1.312 pupils of elementary schools, 8–12 years old) self-reported as victims, 6.25% as bullies and 4.8% as victims and bullies. In the study of Sapouna [40], a lower percentage of students is reported to be involved with bullying. Specifically, 8.2% of the sample (1758 students of elementary and secondary schools) were reported as victims, 5.8% as bullies and 1.1% as bullies and victims. Similar results are reported by another Greek study [41], which was conducted within a sample of 502 students of secondary schools. Specifically, verbal aggression mostly occurred among other forms of aggression, and differences in the frequencies were noted whether the incidents were reported by the bystanders (52.6% of the sample reported verbal aggression), the bullies (7.8% of the sample reported verbal aggression) or the victims (6% of the sample reported verbal aggression).

A cross-national study conducted in 40 countries [42] reported that 41.3% of Greek boys who participated in the study were involved with bullying over two or three times per month either as bullies, as victims or as bullies/victims. Girl’s rate was also extremely high in the same study, as 28.3% of girls in Greece participated in bullying episodes two or three times per month, either as bullies or as victims or as bullies/victims. Another study conducted in a sample of 369 pupils [43] reported a percentage of 22.8% of victims in primary schools.

Another extensive study [44] conducted among Greek adolescents (5.614 adolescents aged 16–18 years) reported that 26.4% of the sample were involved in bullying episodes at least once per month, while 4,1% of the sample were involved once per week.

A more recent study [45] among 1976 Greek adolescent pupils of urban schools mentions that 11% of the sample were victims of bullying, 5% were bullies and 2.4% were victims and bullies.

### 1.3. Aim of the Present Study

The present study aims to estimate the prevalence of bullying in Greek elementary schools and to study the risk factors that lead to bullying episodes based on teachers’ reports. It also intends to enhance our understanding of how aggression is expressed in Greek elementary school settings from teachers’ perspectives. A structured non-standardized questionnaire was developed for the purpose of this study, that was given to teachers in order for them to report bulling episodes observed every day in schools, as well as the bullies’ personality characteristics. According to Crothers and Levinson [46], a number of assessment tools for school bullying exist in literature that target either peers, such as the Peer Beliefs Inventory or Peer Relations Questionnaire (PRQ), or bullies/victims themselves, such as the Self-Rating Questionnaire on Aggressive Behavior (SQAB) or Olweus’s Bully/Victim Questionnaire (OBVQ). It is our belief that our measurement tool, based on the teacher rating instrument that was presented by Dodge & Coie [47] (1987) and adapted in the current study for Greek school settings, is suitable for detecting bullying episodes and the reporting of bullies’ characteristics by Greek teachers, and has the potential to serve as a future early detection tool for teachers.

Teachers deal with students’ conflicts or even violent incidents on a daily basis, either when they survey pupils during break time or when they teach. It should be noted that teachers’ views on this issue are valuable, since they are trusted by pupils of elementary school age and are often the first recipients of complaints or misconduct. Additionally, a great number of victimized children tend to trust their teachers with their bullying experiences [48]. Since the majority of studies in the literature focus on children’s views, the main contribution of the present research is that we study teachers’ assessments of aggressive behavior, as they are considered to be objective external observers of children’s behavior and possess cognitive maturity and pedagogical experience. In similar studies, it has been noticed that there is an agreement between teachers’ perceptions and those of the bullies [49] or their peers [50] regarding aggressive behavior. Additionally, children hesitate to admit and self-report bullying their peers and, thus, according to Rigby [51], few students report high levels of bullying.

## 2. Methods

### 2.1. Procedure

Teachers of elementary schools and kindergartens were asked to answer a structured non-standardized questionnaire. The questionnaire was sent via e-mail to the majority of elementary schools and kindergartens in urban and rural regions of Greece. The participants were first informed about the aim of the study and gave their consent to participate. All teachers completed the questionnaire voluntarily. No personal data of the participants or their pupils were requested. The questionnaire was first approved by the Research Ethics and Ethics Committee of the University of West Attica.

### 2.2. Participants

The questionnaire was answered by two hundred and twenty-one teachers of elementary schools and seventy-one kindergarten teachers (*n* = 292). Teachers reported the bullying episodes and the specific characteristics of the aggressive behaviors that they had witnessed during the school year, as well as the way they dealt with them in their classrooms, via the questionnaire.

### 2.3. Measures

The questionnaire consisted of three sections. The first section concerned mainly demographic information about the class population (e.g., the number of boys and girls, and the number of children in the classroom who had been diagnosed with learning difficulties or who were immigrants). The second section was answered only by teachers who reported that they had witnessed a pupil who repeatedly acted aggressively. It included, in addition the aggressive children’s personal information (gender, age, diagnosis if one existed, origin, family context, academic achievement, use of medication, popularity), 12 statements that describe various types of aggressive behavior (e.g., reactive/proactive aggression, direct/indirect aggression and verbal/physical aggression). In particular, teachers were asked questions such as “How often does the pupil get angry easily and respond when he is teased?” or “How often does the pupil use physical power to dominate his peers?” or “How often does the pupil disobey rules of the classroom and adults’ recommendations?”. These questions were based on the teacher rating instrument that was presented by Dodge & Coie [47]. The answers were given on a five-point scale ranging from 0 to 5 (never, rarely, sometimes, often, and very often), and teachers noted the frequency with which they observed the behaviors of the aggressive child. We should note that the internal consistency of the 12 statements was measured using a Cronbach’s alpha value of 0.758 (Table 1), a value acceptable for our analysis [52]. In the third section, teachers answered how they deal with bullying episodes on a five-point scale. In particular, teachers were asked to evaluate how often they use different methods of intervention for the bullying episodes, such as remarks/punishment given by the teacher or the principal, discussion of the situation in the classroom, reports given to parents or discussions with a school psychologist.

### 2.4. Statistical Analysis

The statistical analyses of the data were conducted using the Statistical Package for Social Sciences 27 (IBM SPSS 27), licensed by the University of West Attica. Descriptive statistics were used to identify aggressive children’s sociodemographic and personal characteristics, which are presented as means (M), medians and standard deviations (SD). Chi-square tests were performed in order to investigate correlations between our categorical variables. For the analysis of the collected data, exploratory factor analysis was used with Varimax Rotation. The extraction method used was Principal Component Analysis (PCA), a technique that is employed to reduce a larger set of variables and to detect underlying associations [53]. Additionally, since the sample size was limited, a Kaiser–Meyer–Olkin (KMO) test, a measure of sample adequacy, was conducted. Bartlett’s test of sphericity was run in order to investigate the adequacy of correlations between the variables [53]. The significance level (*p*-value) was set to 0.05.

## 3. Results

### 3.1. Descriptive Statistics

The percentage of aggressive children in school settings reported by teachers is 70.5% (206 from 292 answers). The frequencies and percentages of aggressive children by age, gender, region and other social and demographic characteristics are shown in Table 2. As we can see, a significant prevalence of boys (86% of aggressive children) is observed. Except for pupils in kindergarten which represent 25.9% of the aggressive children, we notice that there are similar percentages regarding the distribution among different ages. It is also worth reporting that, as we can see in Table 2, a significant percentage of aggressive children (53.1%) have low academic performance. Furthermore 38.2% of the aggressive children are popular among their peers. Finally, we can observe that the percentage of aggressive children reported by teachers is higher in urban Greek schools (56%).

The means, medians and standard deviations for each form of treatment that teachers use when they deal with bullying episodes are shown in Table 3. We should note that the statement about punishment given by the principal refers only to the teachers of elementary schools due to the nature of kindergartens’ school management. Discussion about the bullying episode in the classroom is the most frequently used technique among teachers, while punishments (imposed by the teacher or by the principal) are a less-used technique.

Teachers reported that most bullying episodes occur during breaks in the school yard. The means, medians and standard deviations are shown in Table 4.

The means, medians and standard deviations for each form of violence are shown in Table 5. Cyberbullying was studied only for pupils of elementary schools. This type of bullying is observed less in children of that age, while physical bullying is the most frequent. Finally, the most commonly observed behavior by teachers is the expression of anger among peers.

### 3.2. Correlations

Significant correlations (a < 0.05) found between various forms of aggression and children’s sociodemographic characteristics after conducting chi–square tests are shown in Table 6. In particular, the pupils’ sociodemographic characteristics that were taken into consideration were gender, age, nationality, diagnosis, family context, academic performance, use of medication and popularity. The correlations between any form of children’s aggression and the perpetrator’s nationality, whether they were from a single-parent family or their age are not statistically significant. Receiving medication is also not correlated with any form of aggression.

A pupil’s gender significantly influences their behavior. In particular, gender and physical violence are strongly associated. Boys are more likely to use physical violence towards their peers than girls (*p* < 0.001), have more difficulty in anger management (*p* = 0.007), and become angry more easily and fight back (*p* < 0.001).

Low academic performance and popularity among peers are also factors that influence specific forms of aggression. Pupils’ low academic performance is correlated with the use of physical violence (*p* = 0.018), breaking rules in games (*p* = 0.001) and disobeying teachers’ recommendations (*p* = 0.007).

### 3.3. Factor Analysis

A factor analysis was conducted for the data collected in order to identify the main variables that lead to pupils’ aggressive behavior in elementary schools. The questionnaire given included 12 variables that described different forms of aggressive behavior. The variables are clearly shown in Table 7.

**Figure 1:**
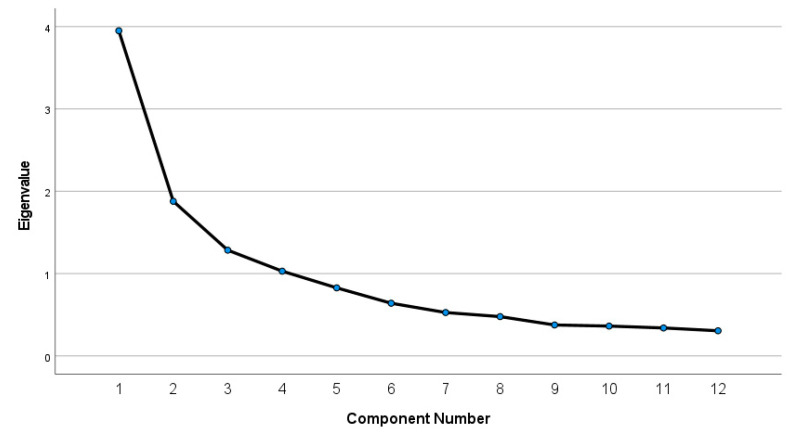
Scree plot depicting the number of components with eigenvalues >1.

Using Principal Component Analysis, four main factors were obtained that had an Eigenvalue > 1, explaining a cumulative variance of 67.86%. A scree plot confirms the findings of the four retained factors (Figure 1).

Communality values that assess how well variables are explained by the factors are depicted in Table 7.

In this study, the KMO was 0.775, which indicates that the sample was adequate for performing factor analysis, and the Bartlett’s Test of Sphericity result was significant (*p* < 0.001), as shown in Table 8.

Table 9 shows factor loadings after using Varimax with the Kaiser Normalization rotation method, with a significant factor criterion of 0.4.

As shown in Table 9, factor 1 is composed of items 5, 2, 6, 1 and 9. These questions focus on variables that can be labeled “offensive behavior” and include leading peers to exclude a pupil from a team activity (item 5), indirect verbal bullying against peers (item 2), threatening and bullying peers (item 6), direct verbal bullying (item 1) and blaming peers in fights (item 9).

Factor 2 consists of items 12, 11 and 3. This factor encompasses variables that can be labeled “misconduct behavior”. It is mainly loaded by item 12 (disobedience of teacher’s recommendations) and item 11 (breaking rules in team games), and to a smaller extent, by the item 3 (physical bullying).

Factor 3 includes three items (10, 8 and 7). These items are related to anger and can be labeled as “outbursts of anger”. It includes item 10 (difficulty in anger management), item 8 (responding negatively when they fail) and item 7 (becoming angry easily and fighting back when teased).

Factor 4 can be labeled “cyber-harassing behavior” and is mainly loaded by item 4, which represents cyberbullying, and to a smaller extent, by items 7 and 9.

**Figure 2:**
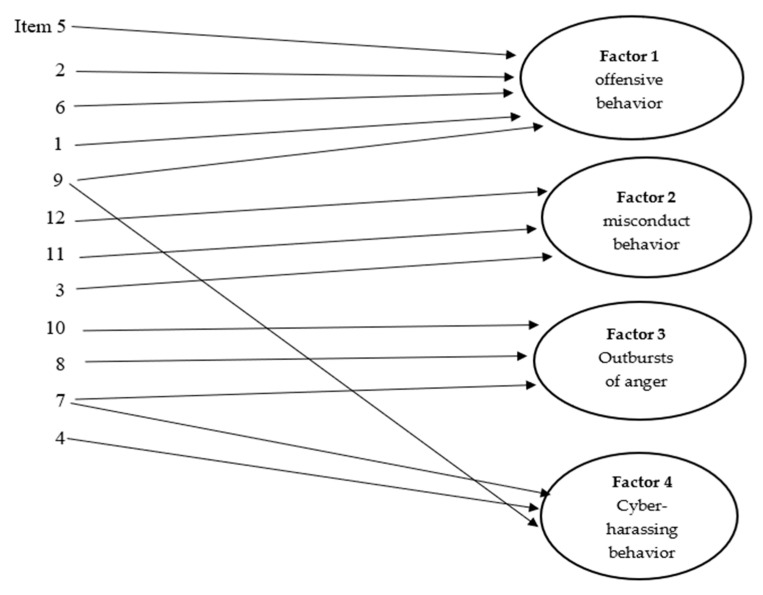
Visual representation of extracted factors.

Finally, a visual representation of extracted factors is depicted in Figure 2, where factor loadings are clearly shown.

## 4. Discussion

The primary purpose of this study was to examine the prevalence of bullying in Greek school settings, from the point of view of teachers of elementary schools; therefore, a structured non-standardized questionnaire was used to outline the aggressors’ characteristics and to evaluate the forms of bullying that prevail in Greek elementary schools. Furthermore, a novel tool for teachers could potentially be developed in order to help teachers in the early detection and recognition of aggressive behaviors.

According to the results of this study, aggressive behavior in elementary school is not associated with the aggressor’s age, nationality or family status, which is in accordance with the results of other studies [23,24,54]. The current research also shows that boys are more likely to be physically aggressive than girls, a result that is also confirmed also by a number of previous studies [2,13,16,19,20,21]. Moreover, according to prior findings [55], bullies tend to have low academic performance, a result that is confirmed by the current study, as well. This is also in accordance with Benbenishty et al. [56], who found that improving academic performance creates a positive school climate, and consequently, prevents incidents of violence.

We should note that in the present study, Greek teachers report that bullying episodes occur more often in the school yard during breaks than during class, a finding that agrees with previous research, since children have more opportunities to receive or initiate an aggressive act [9]. In addition, Vaillancourt et al. [57] reported that pupils of elementary schools involved with bullying episodes feel less secure and vulnerable to aggressive acts in the schoolyard during breaks, where supervision is less strict compared to in the classroom.

Furthermore, according to our findings, Greek teachers prefer to avoid punishment when a bullying episode occurs; they mainly discuss the episode in the classroom, or to a smaller extent, inform both the perpetrator’s and the victim’s parents about the incident. This result is consistent with the findings of a German study that reports that supportive intervention methods, rather than punitive ones, are most frequently used by educators [58].

At this point, we should note that the main novelty and contribution of this study is the understanding of the tendency that exists for Greek pupils to exhibit aggressive behavior in school settings. In particular, through the conducted factor analysis, four dominant factors in the aggressive behavior observed by teachers were revealed. These four factors were labelled: “offensive behavior”, “misconduct behavior”, “outbursts of anger” and “cyber-harassing behavior”, with respect to the main aggressive act that each one represents. A more detailed presentation of the four factors follows.

The first factor, labeled “offensive behavior”, is loaded by both proactive (leading peers to exclude a pupil from a peer activity, and threatening and bullying peers) and reactive (blaming peers in fights) aggressive acts, as well as by direct and indirect verbal bullying. This specific factor is labeled as “offensive behavior” since it describes children who express themselves mainly verbally and insult their peers, either in a direct way or indirectly. Note that the above finding shows that both forms of reactive and proactive aggression can be present simultaneously in bullies in Greek school settings.

The second factor, labeled “misconduct behavior”, refers to pupils who tend to violate school rules and exhibit physically aggressive acts. It includes acts whereby children mainly break rules, either in team play or with their teachers, and it is also loaded, to a lower extent, by physical bullying.

The third factor, labeled “outbursts of anger”, mainly concerns anger expression with or without external provocation. It describes hot-tempered children who have difficulty in anger management, respond negatively when they fail and fight back when they are teased by their peers.

Finally, the fourth factor, labeled “cyber-harassing behavior”, is loaded mainly by cyberbullying and refers to a form of bullying that takes place through the use of electronic devices. This factor also includes two reactive aggressive acts and describes pupils who blame peers in fights, become angry easily and fight back, and thus, tend to be irritated when they are provoked either by educators or their peers.

The factors mentioned above go beyond the distinction of reactive and proactive aggression. This is supported by the fact that teachers reported aggressive acts that are not completely reactive or proactive. On the other hand, the above results clearly support the distinctiveness of verbal and physical aggression, which dominate in the literature.

### Implementations and Limitations

The current literature shows that teachers not only need to be aware of bullying situations in order to respond in the most appropriate way [59,60] but also that they have difficulty in appreciating the seriousness of a conflict [61].The above categorization of aggressive acts deduced from the current study has the potential to provide a novel tool in order for teachers to detect bullying behaviors early, rapidly recognize the nature of aggressive acts and, thus, intervene more effectively; thus, teachers can have better perception of the phenomenon and can create and apply efficient intervention programs. For example, the existence of the factors “outbursts of anger” or/and “cyber-harassing behavior”, both pointing to difficulty in anger management, should prompt teachers to emphasize emotional development and empathy during their teaching, since a number of previous studies have reported the importance of developing empathy in young children in order to prevent school bullying [62,63,64]

As mentioned in the previous section, one dominating factor, according to our results, is “misconduct behavior”. As a result, reducing the tendency to break rules in the school environment will result in a decrease in bullying incidents. Therefore, it is important that teachers be orientated toward developing positive relationships between themselves and pupils in order to reduce misbehavior. The violation of rules could potentially be controlled with strict but fair enforcement of the school rules; this is also supported by other studies where higher disciplinary structure has been associated with lower levels of bullying and victimization [65]. Regarding the limitations of the current study, we should mention that although the sample size was adequate for our research, the results should be interpreted carefully since all teachers are not aware of the bullying forms in school-aged children (especially regarding young ages, i.e., kindergarten grade). Some teachers may misperceive a violent incident and react in a passive way or may have difficulty in recognizing aggressive incidents and the seriousness of the problem in school settings [48]. Moreover, the current survey was based on a self-reported measures and on a convenient sample.

We should also take into consideration that the study was carried out during and after the COVID-19 pandemic, where extreme measures were applied to schools, such as increased supervision and blended learning. Similar studies have shown that school violence rates have decreased in comparison with pre-pandemic conditions [66]. Thus, we should be careful with the generalization of the results and consider the essential educational and social frameworks in which these aggressive behaviors took place.

## 5. Conclusions

This study gives a picture of forms of aggressive behavior dominating in Greek school settings, reported by teachers, and records the prevalence of bullying during and after the crucial period of the pandemic. It is the authors’ belief that this specific perspective has not been reported before. Specifically, four main factors concerning pupils’ violent behavior were revealed: “offensive behavior”, “misconduct behavior”, “outbursts of anger” and “cyber-harassing behavior”. The obtained results contribute significantly to developing a better understanding of bullying problems in Greek schools; therefore, more orientated intervention programs could be created and teachers could recognize and encounter bullying issues more effectively. Based on the obtained findings, a novel tool for the early detection and appropriate intervention in aggressive behavior could be developed, since teachers could potentially adapt their intervention to the dominant form of aggression in their classrooms. Finally, further research should be focused on teachers’ perceptions and awareness of bullying, and could also be extended to pupils of secondary schools in larger-scale studies, combining both quantitative and qualitative methods.

## Figures and Tables

**Table 1:** Reliability statistics of the questionnaire.

Reliability Statistics	Values
Cronbach’s alpha	0.758
Cronbach’s alpha based on standardized items	0.752
Number of items	12

**Table 2:** Sociodemographic characteristics of aggressive children.

Baseline Characteristics	*n*	%
Gender		
Female	28	13.6
Male	178	86.4
Age		
5	53	25.9
6	21	10.2
7	24	11.7
8	28	13.7
9	30	14.6
10	19	9.3
11	30	14.6
Region		
Urban	115	56.0
Rural	91	43,5
Single-parent family ^a^	42	20.3
Foreign children ^a^	34	16.4
Diagnosed children ^a^	55	26.6
Popular among peers ^a^	79	38.2
Low academic performance ^a^	110	53.1
On medication ^a^	16	7.7

Note: *N* = 207 (*n* stands for each condition). ^a^ Reflects the number and percentage of participants answering “yes” to this question.

**Table 3:** Means, medians and standards deviations regarding teachers’ methods of dealing with bullying episodes.

How Teachers Deal with Bullying Episodes	*n*		Mean	Median	Standard Deviation
	Valid	Missing			
Punishment given by the teacher	202	5	2.66	3.00	1.000
Punishment given by the principal ^a^	147	60	2.17	2.00	1.003
Discussion about bullying episode in the classroom	202	5	4.25	4.00	0.786
Giving parents briefing about the incident	201	6	3.73	4.00	1.023
Searching for a specialist’s advice (e.g., psychologist)	202	5	3.12	3.00	1.295

^a^ The statement about the punishment given by the principal refers only to the teachers of elementary schools due to the nature of kindergartens’ school management.

**Table 4:** Means, medians and standards deviations regarding the places that bullying episodes occur most often according to teachers.

Occurrence of Bullying Episode	Mean	Median	Standard Deviation
During class	3.33	3.00	1.056
During intermission	4.01	4.00	0.789

**Table 5:** Prevalence of different types of aggressive behavior.

Forms of Aggressive Behavior	*n*		Mean	Median	Standard Deviation
	Valid	Missing			
1. Direct verbal bullying	206	1	3.36	3.00	1.063
2. Indirect verbal bullying	206	1	2.88	3.00	1.181
3. Physical bullying	206	1	3.56	4.00	0.970
4. Cyberbullying ^a^	153	54	1.46	1.00	0.881
5. Leads peers to exclude a pupil from a team activity	205	2	2.51	3.00	1.132
6. Threatens and bullies their peers	206	1	2.61	3.00	1.115
7. Becomes angry easily and fights back when teased	206	1	4.00	4.00	0.847
8. Responds negatively when they fail	206	1	3.49	4.00	1.121
9. Blames peers in fights	205	2	3.63	4.00	1.137
10. Has difficulty in anger management	205	2	3.89	4.00	1.016
11. Breaks rules in team games	205	2	3.66	4.00	1.034
12. Disobeys teacher’s recommendations	205	2	3.79	4.00	1.010

^a^ This item refers only to the teachers of elementary schools.

**Table 6:** Associations (*p*-values) between aggressor’s sociodemographic characteristics and forms of aggressive behavior after conducting chi-square tests.

	Gender	Age/Class	Nationality	Diagnosis	Single-Parent Family	Low Academic Performance	On Medication	Popular among Peers
Direct verbal bullying	0.598	0.054	0.370	0.170	0.392	0.387	0.228	0.629
Indirect verbal bullying	0.715	0.307	0.417	0.261	0.463	0.409	0.620	0.372
Physical bullying	**0.000**	0.903	0.367	0.064	0.053	**0.018**	0.938	0.207
Leads peers to exclude a pupil from a team activity	0.862	0.816	0.745	**0.025**	**0.043**	0.118	0.465	**0.041**
Threatens and bullies their peers	0.930	0.502	0.759	0.185	0.085	0.772	0.334	0.795
Becomes angry easily and fights back when teased	**0.000**	0.056	0.369	0.422	0.536	0.574	0.072	0.378
Responds negatively when they fail	0.504	0.207	0.212	**<0.001**	0.593	0.376	0.182	**0.017**
Blames peers in fights	0.467	0.809	0.267	**0.001**	0.761	0.752	0.440	0.480
Has difficulty in anger management	**0.007**	0.344	0.369	**<0.001**	0.995	0.286	**0.015**	**0.001**
Breaks rules in team games	0.315	0.255	**0.030**	**<0.001**	0.739	**0.001**	0.153	**0.001**
Disobeys teacher’s recommendations	0.156	0.250	1.000	0.054	1.000	0.007	0.378	**0.041**

Note: *p*-values < 0.05 are noted in bold.

**Table 7:** Communality values.

	Initial	Extraction
Direct verbal bullying	1.00	0.637
Indirect verbal bullying	1.000	0.697
Physical bullying	1.000	0.374
Cyberbullying	1.000	0.745
Leads peers to exclude a pupil from a team activity	1.000	0.681
Threatens and bullies their peers	1.000	0.677
Becomes angry easily and fights back when teased	1.000	0.798
Responds negatively when they fail	1.000	0.645
Blames peers in fights	1.000	0.662
Has difficulty in anger management	1.000	0.777
Breaks rules in team games	1.000	0.693
Disobeys teacher’s recommendations	1.000	0.758

Extraction method: Principal Component Analysis.

**Table 8:** KMO and Bartlett’s Test.

**Kaiser–Meyer–Olkin Measure of Sampling Adequacy**		0.775
**Bartlett’s Test of Sphericity**	Approx. Chi-Square	570,850
	df	66
	Sig.	<0.001

**Table 9:** Rotated Component Matrix.

	Component			
	1	2	3	4
5. Leads peers to exclude a pupil from a team activity	0.791	−0.191		−0.122
2. Indirect verbal bullying	0.780	0.259		−0.147
6. Threatens and bullies their peers	0.778	0.216	0.122	−0.104
1. Direct verbal bullying	0.676	0.260		0.331
9. Blames peers in fights	0.544	0.326	0.142	0.490
12. Disobeys teacher’s recommendations	0.101	0.859	0.101	
11. Breaks rules in team games	0.122	0.779	0.261	
3. Physical bullying	0.186	0.498	0.299	
10. Has difficulty in anger management		0.239	0.848	
8. Responds negatively when they fail		0.289	0.741	−0.104
7. Becomes angry easily and fights back when teased	0.258		0.653	0.552
4. Cyberbullying	0.294		0.121	−0.802

Extraction method: Principal Component Analysis. Rotation method: Varimax with Kaiser Normalization. Rotation converged in 6 iterations.

## Data Availability

The data that support the findings of this study are available from the corresponding author, A.B., upon reasonable request.
